# A modified Blumgart anastomosis with a simple and practicable procedure after laparoscopic pancreaticoduodenectomy: our center’s experience

**DOI:** 10.1186/s12893-023-02221-1

**Published:** 2023-11-16

**Authors:** Guo-Hua Liu, Xiao-Yu Tan, Jia-Xing Li, Guo-Hui Zhong, Jing-Wei Zhai, Ming-Yi Li

**Affiliations:** Department of Hepatobiliary Surgery, Affiliated Hospital, Guangdong Medical University, Zhanjiang, Guangdong 524000 China

**Keywords:** Pancreatic anastomosis, Laparoscopic pancreaticoduodenectomy, Open pancreaticoduodenectomy, Blumgart, Pancreatic fistula

## Abstract

**Background:**

Laparoscopic pancreaticoduodenectomy(LPD) has become the goal of lots of minimally invasive surgical centers in recent years. Postoperative pancreatic fistula(POPF) is still the barrier to attaining the above goal. Thus, improving anastomosis techniques to reduce the rate of POPF has been a hotspot of surgery. Blumgart pancreaticojejunostomy is considered one of the best anastomosis procedures, with low rates of POPF. However, the original Blumgart pancreaticojejunostomy method is not easy for laparoscopic operation. In consequence, we modified a Blumgart pancreaticojejunostomy technique with a simple and practicable procedure and applied to LPD.

**Methods:**

We collected and retrospectively analyzed the perioperative clinical data of patients who underwent modified Blumgart anastomosis from February 2017 to September 2022. The above patients included 53 cases in open pancreaticojejunostomy(OPD) and 58 cases in LPD. After propensity score matching, 44 cases were included for comparison in each group.

**Results:**

After propensity score matching, the average time for pancreaticojejunostomy was about 30 min in the LPD group. The Clinically relevant POPF(CR-POPF) rate was 9.1%. The length of postoperative hospitalization was 13.1 days. Compared with the OPD group, The CR-POPF rate in the LPD group are not significant differences. But the postoperative length of hospital stay was significantly shorter in the LPD group. Besides, there were no other severely postoperative complications between two groups.

**Conclusion:**

The modified Blumgart anastomosis technique applied to LPD in our Center not only has simple and convenient properties but also low rate of CR-POPF. And this method may be a good choice for surgeons to begin to carry out LPD.

**Supplementary Information:**

The online version contains supplementary material available at 10.1186/s12893-023-02221-1.

## Introduction

Pancreaticoduodenectomy (PD) is very challenging surgery because it involves removing the organs of various abdominal areas, complex anatomical relationships around the pancreas, and difficultly organ reconstruction [1]. Furthermore, the high surgical skill requirement of pancreatic anastomosis by laparoscopic way is more challenging for surgeons [2]. So, laparoscopic pancreaticoduodenectomy(LPD) has become the goal of lots of minimally invasive surgical centers in recent years. However, the substantial postoperative mortality of LPD is still a barrier for them. The most important reason of that is postoperative pancreatic fistula (POPF) which often leads to life-threatening complications such as post hemorrhage and abdominal infection [3–5]. Hence, it is very important to find an anastomosis technique that could reduce the risk of POPF. Thus, improving more safe techniques to reduce the rate of POPF has been a hotspot of this surgery. In 2002, Blumgart at Memorial Sloan-Kettering Cancer Center devised an effective method of anastomosis during the process of OPD and received a good clinical efficacy [6, 7]. To date, the Blumgart pancreaticojejunostomy(PJ) is considered one of the safest anastomosis methods after PD [8, 9]. However, the original Blumgart anastomosis procedures are comparatively complicated for surgeons whether in OPD or LPD. Therefore, a great diversity of modified Blumgart PJ emerged in the various pancreatic centers. But, until now, it has not reached a consensus that which kind of modified Blumgart PJ would be optimal in PD or even in LPD.

Due to the above reasons, we applied a novel modified Blumgart technique to LPD when beginning with this surgery according to the good results of our preliminary research [10] in OPD. The article aims to evaluate the application of this method and share the early experiences of LPD in our center.

## Methods and materials

From February 2017 to June 2022, a novel modified Blumgart PJ, which has not been reported, designed by our Biliary pancreatic minimally invasive center has been applied to 63 cases totally in OPD and 65 cases in LPD. To avoid technique bias, the above cases were only included pure OPD and LPD cases, excluding the cases that needed conversion to open or hybrid surgery. All procedures were performed with the same surgical technique by the same surgical team. The perioperative data were prospectively collected, including perioperative parameters such as and surgical outcomes. The former included age, sex, weight, body mass index(BMI), etc. The latter included the time for pancreaticojejunostomy, length of postoperative hospitalization, the rate of postoperative complications such as pancreatic fistula, postoperative bleeding and abdominal infection. We analyzed the discrepancy of patients between OPD and LPD and used propensity score matching to balance the potential confounders between the two groups. After propensity score matching, 44 cases were included for comparison in each group. And the practicability of the modified method was evaluated by comparing the outcomes with the other methods’ from the existing literatures.

### Clinical data

We collected and retrospectively analyzed the patients’ backgrounds and preoperative characteristics (sex, age, BMI, pancreatic texture, pancreatic duct diameter, and histopathological diagnosis, FRS: fistula risk score [11]), intraoperative outcomes(operation time, the time for PJ, intraoperative blood loss, the length of postoperative hospital stay, radicality), postoperative complications such as the rate of Clinically relevant POPF(CR-POPF), the incidence of postoperative delayed gastric emptying(DGE),, and mortality within 90 days after surgery.(Table 1). All patients themself or their relatives signed surgical consent about the possible advantages and disadvantages of OPD or LPD. The study was approved by our institutional review board(The Affiliated Hospital of Guangdong Medical University, ZhanJiang, China, approval number:PJKT2022-036), and the written informed consents were obtained from all subjects.

### Criteria for cases exclusion and inclusion of patients; groups of patients

Patients undergoing OPD and LPD reconstructed with this modified Blumgart PJ between February 1, 2017, and September 1, 2022, were included in this study. Before PD was carried out by Laparoscopic way in our hospital, from February 1, 2017, we began to apply a novel modified Blumgart PJ to PD which were all performed by open operation way. To August 1, 2019, we have finished OPD by this reconstructed way totally 63 cases. And from then on, Our surgical team started to carry out LPD and used this technique during PJ operating process. To September 1, 2022, we have completed the LPD totally 65 cases. In order to avoid technique bias, Patients included this study were only pure OPD and LPD cases. On the other hand, the primary end point of this study was the incidence of CR-POPF in the OPD group compared with that in the LPD group. For the above two reasons, patients undergoing OPD in the first stage were included OPD group, and patients undergoing pure LPD excluding needed conversion to open or hybrid surgery in the second stage were included LPD group. Besides, the cases with poor cardiopulmonary function such as cardiac function grade 2 or above and severe pulmonary dysfunction, which we think could not bear the pneumoperitoneum for a long time, should also be excluded LPD group. Further- more, the cases which needed vein resection or repair were excluded this study. And the cases without comprehensive perioperative data should be excluded this study. Thus, finally, the cases included in this study involved OPD group (53 cases) and LPD group(58 cases).

### PJ technique

After removing the surgical excision, the bleeding of the Pancreatic section should have been coagulated by Bipolar electrocoagulation before the pancreatic reconstruction began. And before performing the modified Blumgart PJ in LPD, we designed the patient’s operative position and the trocar distribution according to the study [12] of Pi-Jiang Sun, etc.

**Fig. 1:**
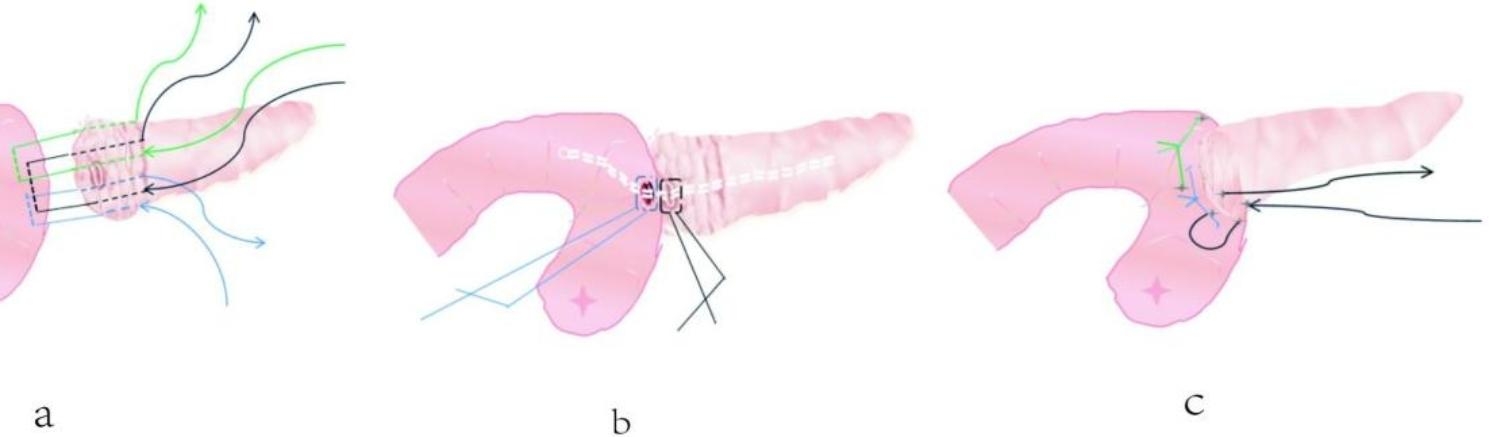
Design sketch of the pancreaticogastrostomy technique. **(a)**: U-shaped suture through the pancreas with intermittent interlocking suture. **(b)**: The pancreatic duct jejunum anastomosis is placed through a stent, and two ends of the stent are purse sutured and folded. **(c)**: Discontinuous interlocking U-shaped suture in the anterior pancreas wall and jejunum serosa

**Fig. 2:**
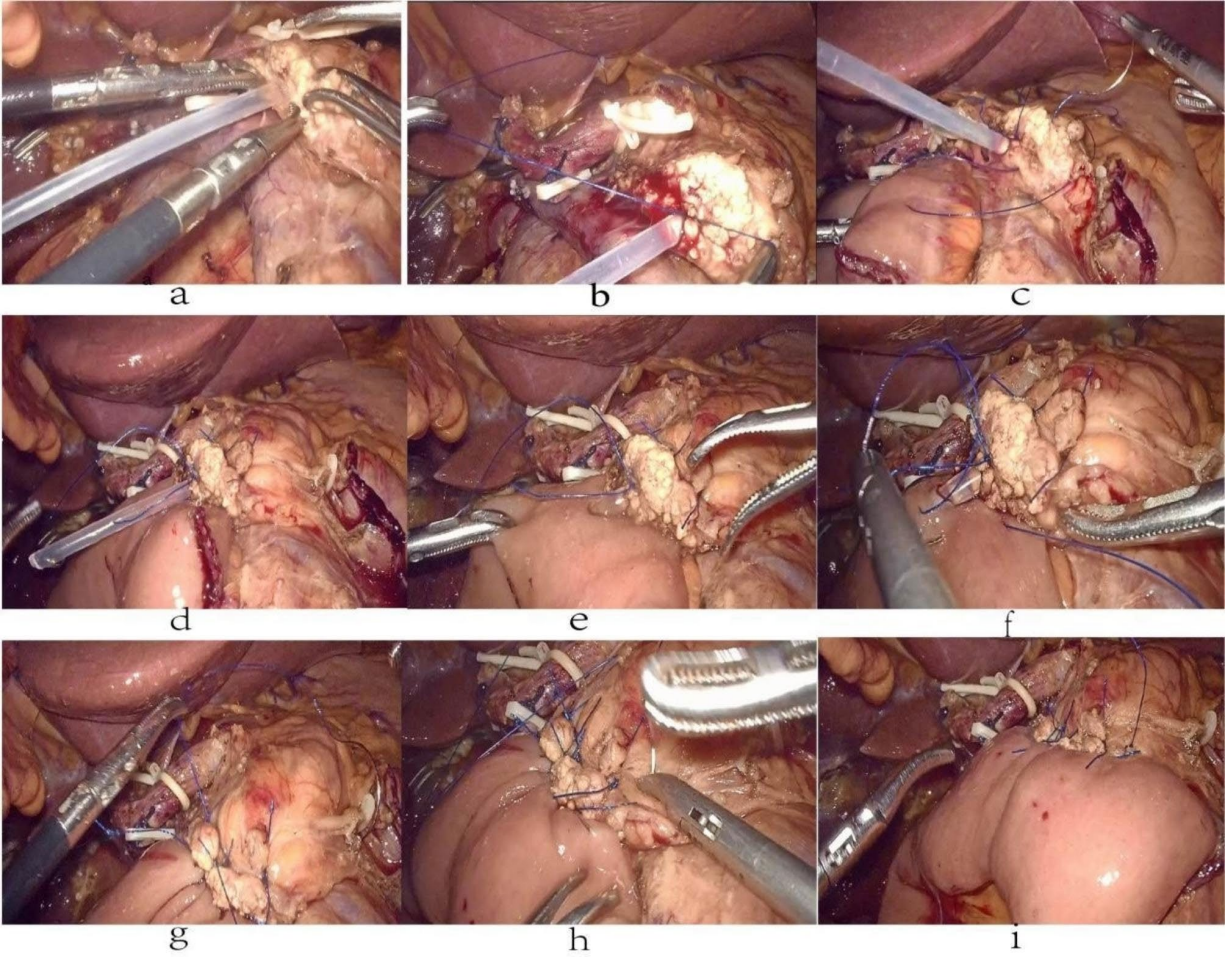
Intraoperative images of the reconstruction of PJ following modified Blumgart method in LPD. **(a)**: A stent tube with a suitable diameter matching pancreatic duct was inserted into the pancreatic duct. **(b)**: The potential gaps between the stent tube and pancreatic tube cavity were closed by purse suture with 4 − 0 Prolene suture line. **(c)**: A 3 − 0 Prolene suture line with a large needle ran through the whole layer of the pancreas at the site 0.5 ~ 1.0 cm from the edge of the pancreatic stump, then entered the seromuscular layer of the jejunal posterior wall. After protruding out, the needle penetrated through the whole layer of the pancreas again, and the breath of suture was 1.0 ~ 1.5 cm. **(d)**: The first layer suture could be finished only needed for 3 ~ 5 stitches which were overlapped. **(e)**: The other end of the stent tube be inserted into the jejunum lumen through a small incision. **(f)**: The potential gaps between the stent tube and jejunum lumen were closed by purse suture with 4 − 0 Prolene suture line. **(g)**: The ends of the above two purse suture ’lines were tied together to form a fixed sinus between the pancreatic duct and jejunum mucosa. **(h)**: The second layer was made a simple interrupted at 3 or 5 sites in the overlapped way or continuous suture according to the width of the pancreas between the seromuscular layer of the jejunal anterior wall and the anterior layer of the pancreatic stump. **(i)**: The image of modified Blumgart PJ finished

Then, the procedure of PJ technique as follows: (1)One end of a stent tube with a suitable diameter matching pancreatic duct was inserted into the pancreatic duct (Fig. 2a), Than, the potential gaps between the stent tube and pancreatic tube cavity were closed by purse suture with 4 − 0 Prolene suture line (Fig. 2b). (2)A 3 − 0 Prolene suture line with a large needle throughout the whole layer of the pancreas at the site 0.5 ~ 1.0 cm from the edge of the pancreatic stump made a U-shaped suture of the seromuscular layer of the jejunal posterior wall for only 3 ~ 5 stitches which were overlapped (Fig. 2c). (3)When the second procedure had been finished, the U-shaped suture lines through the pancreas with intermittent interlocking sutures were knotted together one by one(Figs. 1a and 2d). (4)The other end of the stent tube was inserted into the jejunum lumen through a small incision which was made from the corresponding position on the serosal surface of jejunum stump with an electric coagulated hook(Fig. 2e). The potential gaps between the stent tube and jejunum lumen were closed by purse suture with 4 − 0 Prolene suture line.(Fig. 2f). Then the ends of the above two purse suture lines were tied together to form a fixed sinus between the pancreatic duct and jejunum mucosa(Figs. 1b and 2g**)**. (4) The last step of the PJ is making 3 to 5 U-shaped or 8-shaped suturing in the overlapped way between the seromuscular layer of the jejunal anterior wall and the anterior layer of the pancreatic stump (Fig. 2h). Care should be taken to not damage the pancreatic tissue when each knot were tied (Figs. 1c and 2i).

### Definitions of Complications

POPF was graded according to the new definition and grading system by the International Study Group for Pancreatic Fistula (ISGPF) [13]. Grade A refers to a biochemical fistula, and grades B and C were considered clinically relevant pancreatic fifistulas(cr-POPF) [14–17]. Delayed gastric emptying (DGE)refers to dysfunction of functional emptying of the residual stomach, which usually needs gastrointestinal decompression for more than 7 days [18]. And DGE, Biliary leakage, Abdominal infection, Postoperative hemorrhage(PHH)were identified and classified using standardized criteria as defined by the International Study Group of Pancreatic Surgery (ISGPS) [19–21].

### Postoperative management

All patients were managed with the standard operating procedure of our center for PD. Firstly, the gastric tube was removed on postoperative day (POD) 1 or POD 2 if the gastrointestinal anastomosis bleeding could be excluded. Secondly, the amylase concentration in the drainage fluid of each drainage tube was examined on POD1,3,5,7. Computed Tomography Angiography(CTA) of the upper abdomen was performed on POD7. The drainage tube should be stretched out for 1-2 cm when the volume of drainage was below 50ml. Only if the amylase level of the drainage fluid was lower than 3-fold of the normal blood, the volume of drainage was below 200ml, and simultaneously CTA showed no ascites in the operative regions, the abdominal drains could be removed on the 7th day after surgery. Operative mortality was defined as any death occurring within 90 days of surgery.

### Propensity score–matching strategy

Propensity score matching was chosen in this nonrandomized study for that could balance the potential confounders and, therefore, minimize selection bias when evaluating the application of this method. Individual propensity scores were developed through logistic regression modeling based on the following four covariates: pathology of the disease, pancreatic duct diameter, pancreatic texture, and intraoperative blood loss, which were commonly used in the Callery fistula risk scoring system [22, 23] to predict CR-POPF. Then, the OPD and LPD cases were paired using the exact matching method according with 1:1 ratio and the matches starting from cases with the largest propensity score.

### Statistical analyses

Data analysis in this study was performed using the IBM SPSS 25.0 software. The measurement data are expressed by x ± s with t test; the adoption rate or composition ratio is represented by χ 2 test. When the propensity score was matched, the propensity value of the patient was calculated according to the data of each variable of each patient, and the patient with the closest propensity value was matched 1:1 with a matching tolerance of 0.02. P < 0.05 was considered to be statistically significant.

## Results

### The demographic and clinical characteristic

To September 1, 2022, we have carried out LPD totally 65 cases with this modified Blumgart PJ in our centers since August 2019. Among this above cases, 7 cases converted to open operation were all due to the excessive bleeding or difficulties during tumor resection and no cases conversion were because of difficulties in performing the pancreatic anastomosis. Table 1 shows that the tumor site of LPD’s patients was mainly vater ampulla carcinoma such as Ampullary tumor, Distal bile duct tumor, and duodenal papilla tumor. The average intraoperative blood loss, the rate of R0 radicality were no significant differences while compared with the OPD group. The same results occurred in the other background characteristics and surgical outcomes between OPD and LPD involving age, sex, weight, BMI, hepatic function index, primary tumor origin, pancreatic duct diameter, pancreatic texture. The operation time and the time for PJ had statistical differences while the consuming time were nearly close between OPD and LPD. However, the postoperative length of hospital stay was significantly shorter than that in OPD.

### Postoperative complications

Table 2 shows that no matter OPD group or LPD group, there was a low incidence rate of postoperative complications which involved biliary leakage, delayed gastric emptying, and postoperative pancreatic fistula. No cases were found to be dead within 90 days after the surgery. And the severe complications such as postoperative hemorrhage and abdominal infection didn’t happen in either group. There were not significant differences between POPF of the two groups.

**Table 1:** Background and operative characteristics of the patients

	Before propensity score matching			After propensity score matching		
	OPD	LPD	P value	OPD	LPD	P value
**Patients,n**	53	58		44	44	
**Age,n(%)**	57.1 ± 9.0	58.9 ± 9.7	0.294	57.2 ± 9.0	59.1 ± 9.8	0.360
**Sex,n(%)**			0.558			0.189
Male	30(56.6%)	36(62.1%)		30(68.2%)	24(54.5%)	
Female	23(43.4%)	22(37.9%)		14(31.8%)	20(45.5%)	
**Weight(kg)**	62.8 ± 4.6	62.4 ± 4.7	0.679	63.0 ± 4.4	62.1 ± 4.6	0.357
**BMI(kg/**^**2**^)	21.2 ± 2.3	21.1 ± 2.2	0.941	21.2 ± 2.3	21.0 ± 2.2	0.600
**Hepatic function index**						
ALT	56.6 ± 14.5	61.1 ± 13.1	0.230	55.9 ± 15.9	59.8 ± 12.5	0.151
ALB	32.8 ± 3.9	33.5 ± 2.5	0.402	33.1 ± 2.3	33.7 ± 2.3	0.254
TB	129.0 ± 31.0	128.1 ± 20.4	0.851	129.5 ± 33.3	128.1 ± 20.7	0.821
**Preoperative chemotherapy**			1.000			1.000
Yes	0(0%)	0(0%)		0(0%)	0(0%)	
No	53(100%)	58(100%)		44(100%)	44(100%)	
**Presence of biliary stent**			1.000			1.000
Yes	52(98.1%)	56(96.5%)		43(97.7%)	42(95.5%)	
No	1(1.9%)	2(3.5%)		1(2.3%)	2(4.5%)	
**Pancreatic duct diameter(mm)**			0.954			1.000
≤ 3	18(34.0%)	20(34.5%)		18(40.9%)	18(40.9%)	
> 3	35(66.0%)	38(65.5%)		26(59.1%)	26(59.1%)	
**Pancreatic texture(n,%)**			0.252			1.000
Hard	13(24.5%)	20(34.5%)		13(29.5%)	13(29.5%)	
Soft	40(75.5%)	38(65.5%)		31(70.5%)	31(70.5%)	
**Operative characteristics**						
Operation time (min)	258.7 ± 29.4	299.1 ± 48.0	< 0.001	261.7 ± 30.4	299.7 ± 45.9	< 0.001
PJ-time (min)	27.4 ± 6.4	29.9 ± 4.1	0.017	27.4 ± 6.1	29.9 ± 4.3	0.030
Blood loss(ml)	129.3 ± 60.0	135.2 ± 75.7	0.653	125.8 ± 59.7	133.9 ± 69.1	0.562
**Hospital stay (days)**	16.6 ± 2.5	12.8 ± 2.7	< 0.001	16.9 ± 2.4	13.1 ± 2.9	< 0.001
**Radicality(n,%)**			1.000			1.000
R0	52(98.1%)	56(96.5%)		43(97.7%)	42(95.5%)	
R1/2	1(1.9%)	2(3.5%)		1(2.3%)	2(4.5%)	
**Primary tumor origin(n%)**			0.990			0.975
Pancreatic head tumor	3(5.7%)	4(6.9%)		3(6.8%)	4(9.1%)	
Ampullary adenocarcinoma	23(43.4%)	26(44.8%)		23(52.3%)	21(47.7%)	
Distal CBD adenocarcinoma	6(11.3%)	7(12.1%)		6(13.6%)	5(11.4%)	
Duodenal adenocarcinoma	5(11.3%)	6(6.3%)		5(11.4%)	5(11.4%)	
Other malignancy	4(9.4%)	5(8.6%)		4(9.1%)	4(9.1%)	
Benign lesions	12(22.6%)	10(17.2%)		3(6.8%)	5 (11.4%)	
**FRS**			0.917			0.372
Negligible(0)	2(3.8%)	1(1.7%)		2(4.5%)	1(2.3%)	
Low risk (1–2)	16(30.2%)	18(31.1%)		16(36.4%)	16(36.4%)	
Intermediate risk(3–6)	32(60.3%)	35(60.3%)		26(59.1%)	25(56.8%)	
High risk(7–10)	3(5.7%)	4(6.9%)		0(0%)	2(4.5%)	

BMI: Body Mass Index, ALT: Alanineaminotransferase, ALB: Albumin, TB:Total bilirubin,

PJ time: Pancreaticojejunostomy time, CBD:Common Bile Duct, FRS: Fistula risk score11

**Table 2:** Postoperative complications of the patients

	Before propensity score matching			After propensity score matching		
	OPD	LPD	P value	OPD	LPD	P value
**Patients,n**	53	58		44	44	
**Biliary leakage, n (%)**	3(5.7%)	2(3.4%)	0.918	2(4.5%)	2(4.5%)	1.000
**DGE, n (%)**	2(3.8%)	2(3.4%)	1.000	2(4.5%)	1(2.3%)	1.000
**Abdominal infection, n (%)**	0(0%)	0(0%)	1.000	0(0%)	0(0%)	1.000
**PHH, n (%)**	0(0%)	0(0%)	1.000	0(0%)	0(0%)	1.000
**POPF, n (%)**	7(13.2%)	10(17.2%)	0.556	7(15.9%)	8(18.2%)	0.777
Biochemical leakage	4(7.5%)	5(8.6%)	1.000	4(9.1%)	4(9.1%)	1.000
ISGPS grade B and C	3(5.7%)	5(8.6%)	0.814	3(6.8%)	4(9.1%)	1.000
**Re-operation, n (%)**	0(0)	0(0)	1.000	0(0)	0(0)	1.000
**Mortality**, ***n*(%)**	0(0)	0(0)	1.000	0(0)	0(0)	1.000

PHH: Postoperative hemorrhage, POPF: Postoperative pancreatic fistula

Mortality: Death with 90 days after surgery, DGE:Delayed gastric emptying

ISGPS: International Study Group of Pancreatic Surgery

## Discussion

At present, applying the laparoscopic or robotic (minimally invasive) approach to the various fields of surgery has become a trend. Thus, in recent years, performing LPD or robotic pancreaticoduodenectomy (RPD) became the goal of lots of pancreatic surgeons. In addition, a number of studies have confirmed [24–26] that LPD and RPD both have the advantages of less surgical bleeding, less postoperative pain, and short postoperative recovery time when comparing to OPD. Because of the high requirement for the facility of RPD, even though LPD has been considered one of the most difficult laparoscopic surgery for its complicated operation and life-threatening risk, more and more minimally invasive surgical centers have chosen LPD and succeeded to perform LPD [27–29]. However, it requires the surgeons possessing highly difficult laparoscopic operation abilities. And it is not easy for them to set about LPD. That is mainly due to the following two reasons. One of the reasons is the high incidence rate of CR-POPF that leads to life-threatening complications such as PHH and abdominal infection. Thus, it is crucial to reduce the occurrence of CR-POPF for the pancreatic surgical centers which begin to carry out LPD. The risk factors of POPF include age, BMI, tumor type, pancreatic texture, main pancreatic duct diameter, intraoperative blood loss, PJ technique, etc. [30–33]. Among them, PJ is a key step that could be controlled by surgeons in PD surgery [34]. A study from the University of Pittsburgh in the USA suggested that the improvement of PJ technique could significantly reduce the incidence of POPF [31]. Various studies have shown the safety and feasibility of the Blumgart anastomosis with a low postoperative mortality rate(~ 3%), reoperation rate (~ 7%), and POPF rate (~ 20%), which made the Blumgart anastomosis to be widely used in PD [34–37]. But the original Blumgart PJ method is fairly complicated for laparoscopic operation and the complexity of PJ is the other reason for the pancreatic surgeons not easy to set about LPD.

In consequence, we modified a Blumgart PJ with a simple and practicable procedure which had been proved to be effective in our preliminary research10 in OPD and applied that to LPD. We collected and retrospectively analyzed the clinical data of the patients to value the application of this method. There are studies about original Blumgart show that the rate of CR-POPF is 5 ~ 10% [38, 39] And Toru Kojima etc. reported a modified Blumgart PJ technique applied to OPD with a low rate of CR-POPF [37]. No matter before or after propensity score matching, in our study, the incidence of CR-POPF in the OPD group and LPD group are almost the same as that of Toru Kojima. The above result shows that the CR-POPF rate of our study is acceptable compared to the other studies. On the other hand, the total operating time and PJ consuming time of LPD in our study is about 300 min and 30 min which is lower than that of most other clinical research. For example, a similar study conducted by Toru Kojima [40] shows the total operating time is more than 400 min. The PJ consuming time of LPD in Sun’s study [11] is longer than ours. The above outcomes showed that the method may has some advantages. In addition, the rates of POPF are comparable between OPD and LPD, the severe complications didn’t occur in either group, but the postoperative length of stay was significantly shorter in patients with LPD. And it suggests that the procedure of this method is simple and practicable.

In theory, we may analyze the advantages of the modified Blumgart PJ as follows:

(1) We designed that the potential gaps between stent tube and pancreatic tube cavity or intestinal cavity were closed by purse suture. And this design theoretically could make the leakage of the pancreatic juice from the main pancreatic duct to be lower. Besides, the ends of the above two purse suture ’lines were tied together to make the pancreatic duct and jejunum mucosa close to each other and formed a fixed sinus. This simplified design could also make mucosas of the pancreatic duct and jejunum mucosa to heal with each other. However, the operational procedures of this method are more simple than that of the original or other modified Blumgart PJ. (2) The intermittent interlocking suture between the whole pancreatic layer and the jejunum seromuscular layer can effectively prevent leakage of pancreatic fluid from the accessory pancreatic duct. At the same time, the intermittent interlocking U-shaped suture between the anterior pancreatic wall and the jejunum seromuscular layer can effectively cover the jejunum serosa to the pancreatic stump more closely and avoid the formation of dead space.

Of course, this study only is a retrospective study with a small sample size from a single-center, there may be bias in case selection. Therefore, the clinical value of the modified Blumgart PJ technique still needs to be further evaluated through multi-center prospective clinical studies.

## Conclusion

The modified Blumgart anastomosis technique on PJ for LPD in Our Center not only has simple and convenient properties but also low clinically relevant POPF. It may be favorable to the surgeons who want to set out to perform LPD, because this method is relatively easy and safe. However, to elucidate the clinical benefits of this method, the prospective randomized controlled trials at different centers should be taken in the future.

### Electronic supplementary material

Below is the link to the electronic supplementary material.

Supplementary Material 1

## Data Availability

Data are available from the corresponding author upon reasonable request.
